# Molecular Mechanisms of the Efficacy of Cold Atmospheric Pressure Plasma (CAP) in Cancer Treatment

**DOI:** 10.3390/cancers12020269

**Published:** 2020-01-22

**Authors:** Marie Luise Semmler, Sander Bekeschus, Mirijam Schäfer, Thoralf Bernhardt, Tobias Fischer, Katharina Witzke, Christian Seebauer, Henrike Rebl, Eberhard Grambow, Brigitte Vollmar, J. Barbara Nebe, Hans-Robert Metelmann, Thomas von Woedtke, Steffen Emmert, Lars Boeckmann

**Affiliations:** 1 Clinic and Polyclinic for Dermatology and Venereology, University Medical Center Rostock, 18057 Rostock, Germany; luise.semmler@med.uni-rostock.de (M.L.S.); mirijam.schaefer@med.uni-rostock.de (M.S.); thoralf.bernhardt@med.uni-rostock.de (T.B.); tobias.fischer@med.uni-rostock.de (T.F.); steffen.emmert@med.uni-rostock.de (S.E.); 2 ZIK *plasmatis*, Leibniz-Institute for Plasma Science and Technology (INP Greifswald), 17489 Greifswald, Germany; sander.bekeschus@inp-greifswald.de (S.B.); woedtke@inp-greifswald.de (T.v.W.); 3 Oral & Maxillofacial Surgery/Plastic Surgery, University Medicine Greifswald, 17489 Greifswald, Germany; katharina.witzke@med.uni-greifswald.de (K.W.); seebauerc@uni-greifswald.de (C.S.);; 4 Department of Cell Biology, University Medical Center Rostock, 18057 Rostock, Germany; henrike.rebl@med.uni-rostock.de (H.R.); barbara.nebe@med.uni-rostock.de (J.B.N.); 5 Institute for Experimental Surgery, Rostock University Medical Center, 18057 Rostock, Germany; eberhard.grambow@med.uni-rostock.de (E.G.); brigitte.vollmar@med.uni-rostock.de (B.V.)

**Keywords:** cold physical plasma, plasma medicine, reactive oxygen and nitrogen species

## Abstract

Recently, the potential use of cold atmospheric pressure plasma (CAP) in cancer treatment has gained increasing interest. Especially the enhanced selective killing of tumor cells compared to normal cells has prompted researchers to elucidate the molecular mechanisms for the efficacy of CAP in cancer treatment. This review summarizes the current understanding of how CAP triggers intracellular pathways that induce growth inhibition or cell death. We discuss what factors may contribute to the potential selectivity of CAP towards cancer cells compared to their non-malignant counterparts. Furthermore, the potential of CAP to trigger an immune response is briefly discussed. Finally, this overview demonstrates how these concepts bear first fruits in clinical applications applying CAP treatment in head and neck squamous cell cancer as well as actinic keratosis. Although significant progress towards understanding the underlying mechanisms regarding the efficacy of CAP in cancer treatment has been made, much still needs to be done with respect to different treatment conditions and comparison of malignant and non-malignant cells of the same cell type and same donor. Furthermore, clinical pilot studies and the assessment of systemic effects will be of tremendous importance towards bringing this innovative technology into clinical practice.

## 1. Introduction

For some 20 years, physical plasmas have been used in clinical applications. While thermal (hot) plasmas that are, for example, commonly used in endoscopic tissue coagulation [1] destruction of human tissues, nonthermal (cold) plasmas can be used in clinical applications without harming the treated tissue. Plasma is an ionized gas generated by adding energy in the form of heat or electromagnetic fields to a neutral gas. Such an excited gas contains free charged particles, radicals, UV-radiation, electric fields, and often high temperatures [2]. Plasma treatment generates reactive oxygen and nitrogen species, including O, O_3_, OH, H_2_O_2_, HO_2_, NO, ONOOH amongst many others. According to the current understanding, especially reactive oxygen and nitrogen species (RONS), generated by CAP, induce oxidative damage in the cell, resulting in cell death [3,4,5]. The use of nonthermal plasmas, especially cold atmospheric pressure plasmas (CAP) has been assessed for a variety of different clinical applications including disinfection, wound healing, treatment of atopic eczemas, itch, pain, skin barrier dysfunctions and scars [6]. More recently, the potential use of CAP in cancer treatment has gained increasing attention [7]. In contrast to other applications such as wound healing, the use of CAP in cancer treatment aims at killing the treated tumor cells using prolonged treatment times. In order to understand and improve the efficacy of CAP in cancer treatment it is essential to gain insights regarding the underlying mechanisms of action. Therefore, in this review, we discuss the current understanding of how CAP induces cell death and what factors may contribute to its selectivity towards cancer cells compared to their non-malignant counterparts. First, the immediate effect of plasma components on the treated cells as well as differences between cells that may lead to an enhances sensitivity of cancer cells are discussed followed by a discussion of downstream consequences and signaling pathways that finally induce cell death. Furthermore, we discuss the potential of CAP to trigger an immune response, and thus, its use in combinatorial therapies. Finally, our overview demonstrates how these concepts bear first fruits in clinical applications applying CAP treatment in head and neck squamous cell cancer as well as actinic keratosis.

## 2. Selectivity of CAP towards Malignant Cells

The potential selectivity of CAP towards cancer cells compared to their non-malignant counterparts has enhanced the interest in CAP as an innovative cancer treatment. A review of literature comparing cancer cells to homologous normal cells by Yan et al. revealed that 26 of 33 assessed cell lines showed a strong selectivity, 5 of 33 a weak selectivity, and only 2 of 33 showed a negative selectivity [8]. However, it is important to note, that in this context “homology” had been defined to indicate that cancer cells and normal cells originate from the same tissue type. That means the cells which had been compared in this study have not necessarily been of the same cell type and they didn’t necessarily originate from the same individual. In many cases the cancer cells were cultured in different media compared to the normal cells [9,10,11,12,13,14,15]. However, it is now a well-accepted expectation that a selectivity study should compare malignant and normal cells derived from the same tissue. Furthermore, cells should also be of the same cell type and cultured under comparable conditions. In fact, a recent study has shown, that cell type, cancer type, and culture conditions strongly influence CAP treatment and hence need to be considered when selectivity of CAP is determined [16]. A study comparing a human breast cancer cell line (MCF7) with a normal breast epithelial cell line (MCF10A) showed a dramatically reduced viability of the cancer cells comparted to the normal cells after CAP treatment [9]. However, the two cell lines were not cultured in identical media. The importance of using the same culture medium to test for selectivity was demonstrated elegantly in a study that also compared a human breast cancer cell line (MDA-MB-231) with a normal breast epithelial cell line (MCF10A) [17]. In this study, migration and circularity of the cells were used as proxies for cell viability and functionality. Using a DMEM-based medium, a selective reduced viability was observed in cancer cells compared to the normal cells. However, when using DMEM/F12-based medium no selective effect was observed. Considering the challenges in setting up comparable experimental conditions in order to elucidate a selective effect of CAP on cancer cells compared to their normal counterparts, further studies are required before selectivity of treatment can be claimed. However, differences between malignant and non-malignant cells may explain a potential selective effect. In general, cancer cells seem to be more sensitive to oxidative stress compared to normal cells [8]. One example for the difference of cancer cells and normal cells is the number of aquaporins in the cell membrane—aquaporins are usually more abundant in cancer cells (Figure 1①) [18]. Originally, aquaporins have been identified as water channels [19]. Meanwhile, it could be shown that they also facilitate the transport of free oxygen and nitrogen species, such as hydrogen peroxide as well as other small molecules including carbon dioxide, nitrogen monoxide, ammoniac, urea, and glycerol [20,21]. In the membrane of cells, aquaporins form tetramers with a central pore which functions as a selective filter [8,22]. The diameter of the pore varies among different aquaporins and determines what can pass through the pore. The diameter of aquaporin 1 (AQ1) for example is 2.8 Å and too small to efficiently transport hydrogen peroxide into the cell [21]. The diameter of aquaporin 8 with 3.2 Å instead is significantly larger and hence, sufficient to transport hydrogen peroxide. Although the diameter of aquaporin 1 is relatively small hydrogen peroxide still penetrates faster through this channel than through the lipid double layer of the membrane [23]. So far, aquaporins 1, 3, and 8 are known to be involved in the transport of hydrogen peroxide in mammalian cells [24]. Several experiments have shown increasing oxidative stress due to rising intracellular ROS concentrations caused by increased expression of aquaporins [21,25]. In one study, for example, glioma cells and non-malignant astrocytes were treated with CAP-treated medium (DMEM) [8]. By monitoring the intracellular hydrogen peroxide content over the course of three hours it was shown that the tumor cells accumulated hydrogen peroxide significantly faster compared to the non-malignant astrocytes. Hence, the increased expression of aquaporins in cancer cells compared to their non-malignant counterparts may contribute to an increased sensitivity of these cells to CAP treatment [23].

Besides the expression of aquaporins, the diffusion of free radicals is directly dependent on the amount of cholesterol in the membrane (Figure 1②). Cholesterol is the most abundant lipid in the membrane of animal cells. It accounts for about 50% of all lipids and is of great importance providing membrane stability and fluidity [26,27]. Lipid peroxidation by free radicals (an electron from a lipid gets transferred to a free radical) can result in the generation of pores in the membrane with a size of about 15 Å. These pores are large enough to allow the diffusion of different free reactive species into the cell. A high cholesterol content in healthy eukaryotic cells results in a condensation of membrane lipids and hence provides a barrier against the entry of reactive species such as hydrogen peroxide [28]. In tumor cells, the amount of cholesterol is often reduced compared to healthy cells making them more vulnerable to oxidative stress [8,29,30]. If the intracellular oxidative stress triggered by free radicals exceeds the amount that can be handled by the anti-oxidative defense system apoptosis will be induced through a signaling cascade [31]. By means of computer simulations, the permeation of ROS and RNS across native and oxidized phospholipid bilayers has been investigated and these analyses revealed that the assessed RNS (i.e., NO, NO_2_, N2O_4_) and O_3_ can permeate more easily through both native and oxidized phospholipid bilayers compared to hydrophilic ROS (i.e., OH, HO_2_, H_2_O_2_), indicating their potential importance in plasma medicine [32]. Nitric oxide (NO) regulates posttranslational modifications, S-nitrosation, as well as genome-wide epigenetic modifications that can have both tumor-promoting and tumor-suppressing effects [33]. These effects have been described to be concentration-dependent with low NO concentrations being associated with chemo-resistance, anti-apoptosis, proliferation, metastasis, reduced immune response and angiogenesis while high NO is associated with apoptosis, anti-proliferation, anti-angiogenesis, anti-metastasis, and immune response [34,35]. Interestingly, in blood of breast cancer patients, high levels of NO have been detected and increased nitric oxide synthase (NOS) activity in invasive breast tumors compared to benign or normal breast tissue, suggesting a positive correlation between NO biosynthesis and degree of malignancy [36,37]. Considering these already high levels of NO in cancer cells additional CAP generated RNS may overwhelm the system and switch the NO effect from tumor-promoting to tumor-suppressing.

Regarding the role of the anti-oxidative defense system including NAD(P)H, glutathione, superoxide dismutases, catalases, and peroxidases in preventing the induction of apoptosis this system provides yet another mechanism that may be different between tumor cells and their non-malignant counterparts and, hence, may result in a selective response to CAP treatment [38].

Even though final experimental evidence for the selectivity of CAP towards cancer cells is still lacking several common differences between tumor cells and their healthy counterparts, as outlined above, may explain an increased vulnerability of tumor cells to CAP treatment.

## 3. Pathways Triggered by CAP

With regard to the selectivity of CAP towards malignant cells we have discussed the influence of aquaporins, cholesterol, and the anti-oxidative system on the efficacy of CAP. But what are the precise mechanisms that ultimately lead to CAP-induced cell death? As described above, CAP is not only composed of RONS but also contains further charged particles as well as UV radiation and electromagnetic fields. All these components could play a role and have a synergistic effect. However, studies could show that indirect treatment using CAP-treated medium exerts very similar effects compared to direct CAP treatment [8,39]. Based on such studies it is the current understanding that RONS are the most important component of CAP for its efficacy in killing (tumor) cells [40]. Even though RONS seem to be most important for the efficacy of CAP other components must not be fully disregarded. A comparison of direct and indirect CAP treatment revealed a so far unexplained activation of human pancreas adenocarcinoma cells which renders the cells more sensitive towards RONS [41]. This “activation” may be due to short-lived reactive species or other unknown factors that are not present in CAP-treated medium. However, the cytotoxicity of CAP treatment still seems to be dependent on the CAP originated reactive species. This has been illustrated by eliminating CAP originated RONS using scavengers such as cysteine and catalase which also eliminates the cytotoxicity of CAP treatment [42,43]. In the following sections, we will first look at the most immediate effects of CAP originated RONS on cells and then dive deeper into the known signaling cascades triggered by RONS and what consequences this has for the cells.

As mentioned above, a very immediate effect of RONS on the cell membrane is lipid peroxidation (Figure 1②). This leads to an increased influx of reactive species into the cytoplasm. In the cell the reactive species can now react with different molecules and influence a variety of cellular processes. One important second messenger involved in intra- and extracellular signaling cascades is calcium (Ca^2+^) which plays an essential role in cell life and death decisions. It is well known that there is a close interaction between calcium signaling and ROS signaling [44]. A study investigating the calcium homeostasis in melanoma cells revealed increasing calcium concentration in the cytoplasm after CAP treatment [45]. This increase was also observed in the absence of extracellular calcium, indicating that the added calcium originates from intracellular sources. The main storage of intracellular calcium is the endoplasmatic reticulum (ER) which releases calcium through IP_3_ receptors or ryanodine receptors [46]. Both of these receptors are sensitive to ROS as well as to calcium (Figure 1③). By inhibiting ryanodine receptors the calcium influx into the cytoplasm after CAP treatment was reduced to a minimum, even in the presence of extracellular calcium, indicating that the ER is the main source of the increasing cytosolic calcium [45]. An increase of ROS and a rapid release of calcium from the ER into the cytosol are common features of ER stress [46]. In that respect, it is not surprising that CAP induced ER stress has been observed in yeast as well as in human cells [47,48]. Although the ER and mitochondria have distinct functions, they are physically connected via so called mitochondria-associated ER membranes (MAMs). MAMs allow the exchange of calcium, lipids and metabolites between these organelles (Figure 1④) [49]. ER stress induces a calcium overload in mitochondria and consequently activates mitochondria-dependent apoptosis via release of cytochrome c [50,51]. In line with that, mitochondrial oxidation and membrane depolarization, as well as the induction of apoptosis, has been observed in human lymphocytes after CAP treatment [52]. Such a depolarization of mitochondria membrane potential and thus mitochondria-mediated apoptosis as a consequence of CAP treatment has also been observed in human cervical cancer HeLa cells [43].

While the role of ROS has been investigated fairly well, relatively little is known regarding the impact of RNS. The relatively high permeation probability of RNS might contribute to the induction of mitochondrial apoptosis by disrupting the cytochrome c function. Nitric oxide (NO) for example binds cytochrome oxidase, the terminal enzyme of the electron transport chain in mitochondria [53]. Although the mechanisms by which NO exerts its cytostatic/cytotoxic or tissue-damaging effects are not entirely clear, blocking the cytochrome oxidase results in increased levels of intracellular ROS followed by the induction of mitochondrial apoptosis.

Besides the interaction between ROS and calcium signaling, an increase in ROS is also associated with the induction of DNA lesions. Such lesion include oxidative damage, DNA single strand and DNA double strand breaks (DSB) [54,55,56], as well as DNA crosslinks and crosslinks between DNA and proteins [57]. Furthermore, free radicals can cause modifications of purine and pyrimidine rings, strand cleavage and chromosomal abnormalities [58,59,60]. DNA damage as a consequence of CAP treatment has been shown in several studies. However, in these studies primarily DSB have been assessed by detection of γH2AX, a phosphorylated form of the histone H2AX. Phosphorylation of H2AX serves as a well-established indirect marker for DSB (Figure 1⑤). The induction of DSB by CAP is dependent on the distance of the CAP source to the cells as well as on the treatment time [61]. While 30 s treatment led to DSB in 60% of oral cancer cells, treatment for 120 s induced DSB in 80% of the cells. Similar findings have been reported for glioblastoma cells which showed increased DSB after 180 s treatment [62]. Interestingly, this increase in DSB was first detected 72 h post-treatment. A multiphase cell cycle arrest associated with DSB and a subsequent apoptosis induction was also observed in glioblastoma and colorectal carcinoma cells [42]. Here the DSB have been detected three hours after CAP treatment. Likewise, DSB have been observed three hours after treatment in mouse melanoma cells [63]. Since H2AX is phosphorylated by the ataxia telangiectasia mutated (ATM) kinase it is not surprising that an increased expression of this kinase has been observed after CAP treatment in oral cavity squamous cell carcinoma cells [64]. Also, activation of other substrates of ATM involved in signaling apoptosis such as p53 and p73 has been observed in oral cavity squamous cell carcinoma and melanoma cells [64,65,66]. While it has been shown that apoptosis can be induced by ROS through calcium signaling and alteration of the mitochondrial membrane potential (described above) the importance of ATM for apoptosis induction in response to DNA damage has been shown by small interfering (siRNA) knockout experiments. Knockout of ATM resulted in a significant reduction of apoptosis in squamous cell carcinoma cells [64]. Despite these findings, DSB may not be a direct effect of CAP mediated low-ROS on DNA but rather a consequence of CAP induced apoptosis. Blocking apoptosis and p38 MAPK signaling abolished increased γH2AX after CAP treatment in human lymphocytes while UV induced γH2AX was independent of apoptosis [67]. Cell death and growth arrest caused by CAP treatment will be discussed in more detail further down.

Reactive species are not only produced by external sources such as CAP but are also normal by-products of cellular metabolism. In order to counteract oxidative stress and hence prevent the formation of DNA lesions and the induction of apoptosis, cells have evolved a defense system against oxidation (Figure 1⑥) [68]. An important role in this intracellular antioxidant system play thiols by protecting against oxidative and free radical damage [69]. The most abundant intracellular thiol is glutathione (GSH) [70,71]. The amount of GSH increased after CAP treatment in T lymphocytes and the GSH was also significantly oxidized by the treatment [52]. Similarly, plasma treatment decreased the ratio of glutathione to glutathione disulfide (GSH/GSSG) and NADPH/NADP^+^ in cancer cells [72,73]. The oxidation from GSH to GSSG is catalyzed by glutathione peroxidases. *N*-acetyl-cysteine (NAC), a precursor of intracellular GSH is widely used as a scavenger and has been shown to effectively inhibit the increase of intracellular ROS in CAP-treated cancer cells [43,74]. Furthermore, the addition of pyruvate to the culture medium significantly suppressed ROS levels in a lung adenocarcinoma cell line [39]. Besides glutathione peroxidase the intracellular antioxidant system also includes further enzymes such as catalase (catalyzes the decomposition of hydrogen peroxide to water and oxygen) and superoxide dismutase (catalyzes the dismutation of the superoxide) [75,76]. The activity of these enzymes was significantly reduced in HepG2 cells after CAP treatment [72]. Interestingly, the activity of superoxide dismutase was reduced after high dose plasma, but slightly increased after low dose plasma. Modulating ROS levels or targeting antioxidants for cancer treatment is not a new concept. Significantly increasing ROS levels for example is also the basis for the anti-tumorigenic effect of chemotherapeutics such as cisplatin, carboplatin, and doxorubicin [77,78]. To maintain high ROS levels that allow pro-tumorigenic signaling pathways to be activated without inducing cell death many cancer cells are dependent on an increased antioxidant system [79]. Taken together, the cell possesses a comprehensive antioxidant system to protect against oxidative stress but if this system is overwhelmed by CAP generated reactive species the capacity of the different players in the system is limited and consequently cell death is induced.

Another consequence observed after CAP treatment is reduced adhesion, migration, and invasion (Figure 1⑦). Integrins are adhesion molecules on the surface of cells and play an important role in these processes. A significant detachment from the cell culture vessel as well as inhibited expression of integrin α_2_, integrin α_4_ and the focal adhesion kinase (FAK) has been observed in melanoma cells after CAP treatment [80]. Likewise, reduced migration and cell detachment in conjunction with reduced expression of integrin β_1_ and integrin α_v_ have been observed in primary fibroblasts and mouse epithelial skin cancer cells (PAM) following CAP treatment [81]. Although these studies revealed an association of CAP treatment with reduced expression of several integrins, the exact mechanisms leading to the inhibition of integrins still remain to be elucidated. Nonetheless, the inhibition of integrins may be relevant for the efficacy of CAP in cancer treatment since integrins are known to play a crucial role in malignant transformation, inhibition of apoptosis, and the ability to metastasize [82,83].

## 4. Induction of Cell Death by CAP

As described above, CAP can affect several intracellular signal transduction pathways which in turn determine the fate of the cell and may trigger cell death. As a consequence of CAP treatment necrosis or apoptosis may be induced but also the induction of senescence as well as autophagy have been observed (Figure 1⑧). Which of these processes is induced seems to be dose-dependent. While senescence, a well-known irreversible growth arrest in response to stress such as oxidative stress and DNA damage [84], may be induced by relatively short treatments with CAP, apoptosis, and necrosis are induced by prolonged treatment times. For example, melanoma cells treated with higher doses (≥15 s at 1.4 W/cm^2^) using a Floating Electrode Dielectric Barrier Discharge (FE-DBD) plasma source died through necrosis, while very low doses (5 s at 0.8 W/cm^2^) induced apoptosis in these cells [85]. Interestingly, even the higher doses used in these experiments were still below the threshold of damaging healthy tissue [86]. Also using a plasma jet, a treatment time and gas mixture dependent induction of necrosis or apoptosis has been observed in V79-4 cells (normal fibroblasts isolated lung tissue of a Chinese hamster) [87]. Highlighting a difference between primary cells and cell lines, Hirst and colleagues observed necrosis and autophagy in primary prostate epithelial cells and apoptosis and necrosis in cell lines [88]. A predominantly non-accidental form of necrosis due to the interaction of CAP with the extracellular environment was observed by treatment of normal primary fibroblasts using a Helium Guided Ionization Waves (He-GIW) device [89]. Another study showed DNA fragmentation followed by the induction of apoptosis after treatment of head and neck squamous cell carcinoma cells using the Surface Micro Discharge (SMD) plasma technology [90]. Besides necrosis, apoptosis and autophagy also senescence has been observed after CAP treatment [65]. This induction of senescence by SMD generated CAP in melanoma cells is dose-dependent and depends on cytosolic influx of calcium [45]. While sub-lethal doses of CAP in this setting induced senescence, higher doses resulted in the induction of apoptosis [65]. Taken together, different modes of growth arrest and cell death have been observed as a consequence of CAP treatment. While various studies show a clear dose dependency, other factors such as cell type and plasma source may also influence the outcome. Further studies are required to decipher the exact molecular mechanisms and decision points that determine which of these processes will be induced in response to a disturbed redox balance caused by CAP treatment.

## 5. CAP Interaction with the Tumor Microenvironment

In order to understand the effect of plasma, not only the interactions between plasma and the tumor cell itself are important, but also the relationship to the tumor microenvironment (TME). The tumor microenvironment plays an important role in cell survival, growth, invasion- and metastasis of the tumor cells. Furthermore, the TME plays a crucial role for the efficacy of various chemotherapies [91]. Effects of CAP have been observed on different parts of the TME, which is composed of malignant cells, immune cells, endothelial cells, fibroblasts, tumor vasculature and the extracellular matrix, which are in constant communication with each other. In addition to the various cell types, the TME consists of collagen, elastin, fibronectin, glycoproteins, and proteoglycan [92]. It has been observed that prolonged treatment with CAP inhibits cell viability and collagen production of murine fibroblasts [93]. A reduction in collagen secretion and the migration behavior was also observed after CAP treatment in keloid fibroblasts, which, like tumor-associated fibroblasts, show an overproduction of collagen [94,95]. Moreover, in vitro studies have shown that CAP is able to destroy collagen [96]. Eisenhauer and colleagues showed that high doses of CAP prevent extracellular matrix interactions with cells and bone formation [97]. The desmoplastic reaction that has already been shown in the clinical use of CAP for the treatment of head and neck cancer also suggests an increased deposition of collagen [98,99]. Other components of the extracellular matrix, such as hyaluronic acid or fibronectin, can also be damaged or influenced by ROS, although the relationship to CAP has not been sufficiently investigated [100,101]. Particular attention is paid to the effect of plasma on the communication between cells but also between cells and the extracellular matrix and the influence on this communication by treatment with plasma. Some cells sustain damage from plasma treatment even though they are not treated directly. This may be explained by communication between the cells. The bystander effect enables cells to send signals to untreated neighboring cells. Therefore, soluble molecules such as chemokines or growth factors and different junctions can be used. The oxidative stress caused by plasma treatment, influences or damages these signaling molecules [102,103,104]. Alternatively, apoptosis may occur in neighboring cells due to the formation of secondary oxygen and the inactivation of the membrane-bound catalase [105,106]. It has also been shown that calcium ions can be transported from apoptotic to non-apoptotic neighbour cells via gap junctions, which also explains the widespread effect of plasma [107]. A comprehensive review on CAP effects regarding numerous other parts of the TME was provided by Privat-Maldonado et al. [92].

## 6. Induction of an Immune Response through CAP Treatment

The Nobel Prize for Medicine or Physiology 2018 for checkpoint cancer immunotherapies has highlighted the importance of the immune system as a critical contributor to target tumor cells [108]. Because plasma treatment is a local therapy possibly modulating the tumor microenvironment, several reports have addressed the possibility of plasma to stimulate immunity to possibly support anticancer treatment [109,110]. Two lines of research are currently pursued to disentangle the effect of plasma treatment in anticancer immunity. One is the ability of plasma to affect immune cells directly, which leads to their activation or selection of specific subpopulations of immune cells, for example [111]. The second is an indirect activation of immune cells via plasma-mediated tumor cell death and pro-inflammatory signals in the microenvironment [109,110].

Cellular immunity is comprised of innate and adaptive immune cells. While the former recognize evolutionarily conserved epitopes on target structures, the latter can diversify their receptor repertoire to respond to new or mutated antigens. Phagocytes, such as neutrophils, dendritic cells, and macrophages are some of the primary cell types shaping innate immune responses [112]. Macrophages are present in virtually all types of tissues and essential in shaping the local balance of inflammation and anti-inflammation [113]. Plasma-treated cell line-derived macrophages were shown to have a higher migratory activity [114], cytokine release [115], and augmented antitumor toxicity [116], which contributed to elevated levels of TNFα [117] when investigated in transwell co-culture systems. Moreover, plasma treatment was suggested to modulate the differentiation patterns of primary murine [118] and human monocyte-derived macrophages [119]. Using human cell line-derived macrophages, this change in differentiation was also attributed to enhanced antitumor effects in direct co-culture experiments [102]. In Vivo, elevated levels of macrophages were found in pancreatic cancer tissue in response to therapeutically active plasma-conditioned liquids [120]. For neutrophils, there is increasing evidence that their increased presence in tumors and blood is associated with poor prognosis in cancer patients [121]. To date, there is only a single report on plasma-treated neutrophils that describes elevated neutrophil extracellular-trap (NET) formation in response to gas plasma treatment [122]. In mild contradiction to that, evidence of increased intracellular neutrophils and NET formation was found in pancreatic cancer subjected to plasma-conditioned liquid [123], which was associated with survival benefit in these mice. For other innate immune cells, such as NK cells and mast cells, there have been no reports in the context of cancer immunology. For primary NK cells, it is known only that they are similarly sensitive to plasma-induced cell death compared to adaptive lymphocytes, while activated NK cells are less prone to plasma-mediated apoptosis [124]. Similarly, only very few reports have reported response of cells of the adaptive immune system with regard to activation putatively important to anticancer immunity. While activated primary T-cells were also found less sensitive to apoptosis following exposure to plasma [124]. Interestingly, T-cells actively counteract plasma-mediated oxidative stress [52] while increasing markers associated with their activation such as CD69 and HLA-DR [125]. For both innate and adaptive immune cells, plasma treatment regulated the protein content of microparticles released from these cells [126], with microparticles being a biological entity increasingly recognized in cancer research [127].

Extensive plasma treatment times or energies damage tumor cells. There is increasing evidence that such oxidation-induced cell death takes place in a pro-immunogenic manner. The paradigm of immunogenic cancer cell death (ICD) predicts that tumor antigens presented in an immunogenic but not tolerogenic context orchestrate antitumor T-cell responses [128]. If tumor cell death comes with enhanced levels of damage-associated molecular patterns (DAMPs, such as ATP [129]) being paralleled by an increased uptake of tumor material via dendritic cells (DCs, via, e.g., calreticulin; CRT), the latter present tumor antigen to antitumor T-cells together with sufficient T-cell co-stimulation in the draining lymph node [130]. The activated T-cells proliferate and later reach the tumors and their metastases throughout the whole body via the blood. Within the tumor microenvironment, they recognize tumor antigens and lyse the target cells, helping the body to fight cancer using its endogenous weapons provided by the immune system. Using direct plasma treatment or plasma-conditioned liquids, ICD has been observed in vitro in a number of tumor cell types including, for instance, pancreatic cancer, colorectal cancer, lung cancer, and malignant melanoma [99,131,132,133,134,135,136,137,138,139,140]. Due to the extensive poly-pragmasia of plasma sources used in the field of plasma medicine, the central mechanisms underlying plasma-induced ICD have not been commonly unraveled. One of the sources initiating ICD is a dielectric barrier discharge used by Lin et al. The authors elegantly demonstrated a strong dependence of ICD on short-lived reactive species with an only minor contribution of other plasma effectors [131]. However, the exact types of the main species being critical for plasma-induced ICD that would allow optimization of an anti-cancer plasma source specifically targeting ICD pathways were not identified. Moreover, the plasma source is not accredited as medical device, hampering translational efforts of this innovative therapy. For the accredited plasma medical device kINPen, clinical evidence has been reported in the therapy of stage IV head and neck cancer patients [99,141,142,143]. A role of any enhanced immune-mediated effects in this treatment is suggested but not clearly demonstrated yet [98]. However, preclinical animal models suggest involvement of anticancer immunity. An increase of intratumoral T-cells was observed in plasma-treated melanoma [144] and pancreatic cancer exposed to plasma-condition liquid [123]. In the latter, an increase of CRT expression was observed in tumors, which was also found in a model colorectal cancer subcutaneously injected into the skin of mice [135]. In this model, the authors also reported an increase of intratumoral CD11c^+^ expression, indicative of DCs. In addition, van Loenhout and colleagues recently reported increased activation of DCs co-cultured with tumor cells exposed to plasma-conditioned liquid [134]. All these data suggest that plasma treatment of tumor cells shapes antitumor immunity, although the extent of such an effect is subject to further research.

## 7. Clinical Application of CAP

While in vitro studies using cell cultures and in vivo studies using mouse models indicate a huge potential of CAP for cancer treatment, the efficacy ultimately has to be proven for human patients in a clinical setting. First experiences have been reported from treating locally advanced head and neck cancers in six patients [98,99]. Using a plasma jet (kINPen MED) these patients have been treated within one week in three cycles of single applications. This treatment resulted in improved quality of life through a reduction odor and pain medication demands. Two patients showed a partial remission for at least nine month and biopsies from tissues in remission revealed a moderate amount of apoptotic tumor cells. Similar results have been reported in a second study including 12 patients [99]. Analyses of resected CAP-treated tumor tissue revealed an increase of apoptotic cells compared to non-treated tissue [143]. Another case series elucidated the effect of CAP on actinic keratosis (precursor lesions of squamous cell carcinomas) [145]. In this study, a total of 17 lesions have been treated. Nine lesions showed total remission, three a partial remission and only five lesions showed only minimal or no improvement one month after CAP treatment. Of note, no negative effects have been reported. No inflammation, pain, or other adverse events have been observed neither during treatment, immediately after treatment nor in the later course of the disease. Even though more patients need to be treated more than 70% of these patients responded to the therapy [145]. In a second study including seven patients with actinic keratosis, all patients showed a good response with a significant remission of the actinic keratosis after seven treatments for 120 s using a plasma jet [146]. A pilot study including eight patients with malignant pleural mesothelioma investigated the use of cold plasma for cold plasma coagulation (CPC) [147]. CPC was performed as part of a multimodal therapy and the results indicate CPC to be a safe technique when used on the pleura, pericardium, and diaphragm. Histological examinations of pleural specimens revealed no detectable vital tumor cells in deeper layers of the pleural and subpleural space. No relapse of the disease was observed during the time of the study (median observations time was one year). These first clinical reports are very promising (summarized in Table 1), but, of course, can only be the beginning of further clinical trials.

## 8. Conclusions

Reactive oxygen and nitrogen species (RONS) have been identified as the main contributors for the efficacy of CAP in killing cancer cells. Although many studies indicate a selective effect of CAP towards malignant cells compared to their healthy counterparts the experimental settings in many of these studies may have influenced this finding. Nevertheless, several factors have been identified that often differ between healthy and malignant cells and hence, may contribute to an increased sensitivity of cancer cells to CAP. These factors such as expression of aquaporins or cholesterol or the ability to protect against oxidative stress by the anti-oxidative system determine how many RONS can enter the cell and interfere with intracellular signaling pathways. As a consequence of the CAP treatment reduced adhesion, migration and invasion may contribute to a successful cancer treatment by reducing the ability of the cells to spread and form metastasis. Furthermore, necrosis, apoptosis, senescence, and autophagy may result from CAP treatment in a dose-dependent manner and hence, stop tumor growth and trigger an immune response. The underlying mechanisms that decide which process of growth arrest or cell death as a consequence of CAP treatment is triggered still need to be further elucidated. Moreover, the different plasma sources and treatment conditions as well as cell types and tumor entities investigated contribute to the efficacy and always need to be considered when drawing any conclusions. In the end, great progress has been made to the understanding of underlying mechanisms regarding the efficacy of CAP in cancer treatment, but much still needs to be done with respect to different treatment conditions and comparison of malignant and non-malignant cells of the same cell type and same donor. First clinical case reports support the benefits of CAP as a potential innovative therapy for the treatment of cancers and should motivate further clinical trials to prove the relevance of CAP in the clinic.

## Figures and Tables

**Figure 1:**
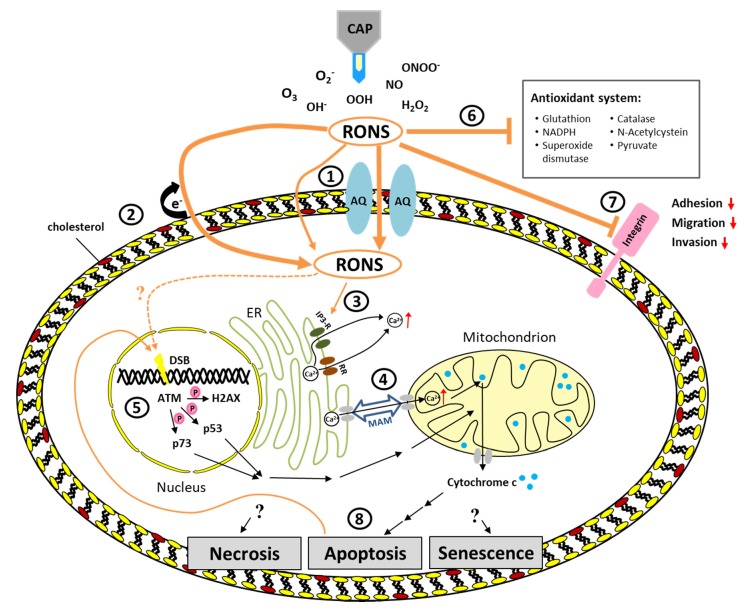
Overview of the current understanding of molecular mechanisms involved in the efficacy of cold atmospheric pressure plasma (CAP) in cancer treatment **①** Aquaporins (AQ), often increased in cancer cells, facilitate transition of reactive oxygen and nitrogen species (RONS) into the cell, while minimal amounts may also diffuse through the cell membrane. **②** Lipid peroxidation by free radicals leads to pore formation in the membrane and hence facilitates diffusion of reactive species into the cell. This effect may be enhanced in cancer cells due to reduced levels of cholesterol-a lipid important for providing membrane stability and fluidity. **③** Increased intracellular RONS interfere with calcium signaling (e.g., through interaction with inositol trisphosphate receptor [IP3-RR] and ryanoid receptor [RR]) resulting in increased calcium influx into cytosol. **④** Furthermore, RONS induced endoplasmic reticulum (ER) stress leads to a calcium influx into mitochondria reducing the membrane potential and hence inducing mitochondria-dependent apoptosis. **⑤** CAP induced DNA double strand breaks (DSB) cause a DNA damage response including activation of ATM, H2AX, p53, and p73. These DSB may not be a direct effect of CAP on DNA but rather a consequence of CAP induced apoptosis. **⑥** Increased levels of RONS produced by CAP overwhelm the antioxidant system and hence limit its protective effect against oxidative stress. **⑦** Reduced expression of integins after CAP treatment may explain the reduction of adhesion, migration, and invasion after CAP treatment. **⑧** As a consequence of CAP treatment necrosis, apoptosis, and senescence have been reported. Which of these processes is induced seems to be dose-dependent. However, the underlying mechanisms that decide which process of growth arrest or cell death as a consequence of CAP treatment is triggered still need to be further elucidated. MAM = mitochondria-associated ER membranes.

**Table 1:** Clinical studies reporting the use of CAP for treatment of (pre-) cancerous tissues.

Reference	Number of Patients	Tumor Entity	Plasma Source	Main Observations after CAP Treatment
Metelmann et al. 2018	6	Locally advanced head and neck cancers	kINPen MED	Improved quality of life due to reduced odor and pain Partial remission in 2 patients
Metelmann et al. 2015	12	Advanced squamous cell carcinoma of the head and neck	kINPen MED	Decreased request for pain medication Reduction of typical fetid odor Reduction of microbial load Superficial partial remission of tumor in 4 patients Wound healing of infected ulcerations tumor in some cases
Schuster et al. 2016	Group I: 12 Group II: 9	Advanced squamous cell carcinoma of the head and neck	kINPen MED	Increase of apoptotic cells in CAP-treated tissue compared to non-treated tissue
Friedman et al. 2017	5 (17 lesions)	Actinic keratosis	Custom-made device with hand-held electrode (FPG10-01NM10)	Total remission of 9 lesions, partial remission of 3 lesions, minimal or no improvement of 5 lesions
Wirtz et al. 2018	7	Actinic keratosis	Adtec Steri-Plas	Number of lesions decrease in 6 of 8 treated areas
Hoffmann et al. 2010	8	Pleural mesothelioma	CPC 1500 System (jet)	No detectable vital tumor cells in the tissue after treatment
